# The Role of Conserved Waters in Conformational Transitions of Q61H K-ras

**DOI:** 10.1371/journal.pcbi.1002394

**Published:** 2012-02-16

**Authors:** Priyanka Prakash, Abdallah Sayyed-Ahmad, Alemayehu A. Gorfe

**Affiliations:** 1 Department of Integrative Biology and Pharmacology, University of Texas Health Science Center at Houston, Houston, Texas, United States of America; 2 Center for Membrane Biology, University of Texas Health Science Center at Houston, Houston, Texas, United States of America; University of Houston, United States of America

## Abstract

To investigate the stability and functional role of long-residence water molecules in the Q61H variant of the signaling protein K-ras, we analyzed all available Ras crystal structures and conformers derived from a series of independent explicit solvent molecular dynamics (MD) simulations totaling 1.76 µs. We show that the protein samples a different region of phase space in the presence and absence of several crystallographically conserved and buried water molecules. The dynamics of these waters is coupled with the local as well as the global motions of the protein, in contrast to less buried waters whose exchange with bulk is only loosely coupled with the motion of loops in their vicinity. Aided by two novel reaction coordinates involving the distance (*d*) between the C_α_ atoms of G60 at switch 2 and G10 at the P-loop and the N-C_α_-C-O dihedral (*ξ*) of G60, we further show that three water molecules located in lobe1, at the interface between the lobes and at lobe2, are involved in the relative motion of residues at the two lobes of Q61H K-ras. Moreover, a *d/ξ* plot classifies the available Ras x-ray structures and MD-derived K-ras conformers into active GTP-, intermediate GTP-, inactive GDP-bound, and nucleotide-free conformational states. The population of these states and the transition between them is modulated by water-mediated correlated motions involving the functionally critical switch 2, P-loop and helix 3. These results suggest that water molecules act as allosteric ligands to induce a population shift among distinct switch 2 conformations that differ in effector recognition.

## Author Summary

K-ras belongs to the Ras family of G-proteins that regulate cell proliferation and development. To execute its function, K-ras adopts different conformational states when it is active and inactive. In addition to these two states, it samples many transient intermediate conformations as it makes the transition from one state to the other. Mutations that affect the population of these states can cause cancer or developmental disorder. Using simulation approaches, here we show that a number of water molecules buried within the structure of an oncogenic K-ras protein modulate the distribution of its conformational states. Moreover, a detailed analysis based on two novel structural parameters revealed the existence of long-range water-mediated interactions that facilitate a dynamic coupling between the two lobes of the protein. These findings pave the way for a dynamics-guided strategy to inhibit abnormal Ras signaling.

## Introduction

The role of solvent on the structure and function of proteins has been the subject of numerous previous studies [1]–[6]. For instance, it has been shown that waters in the dimer interface of hemoglobin play an important role in its transition between deoxy and oxy forms [7], [8]. Water molecules serve as “lubricants” to facilitate conformational inter-conversion [9], [10] or as “adhesives” in binding interfaces [11]. Dehydrated biomolecules therefore lose their biological activity and have suppressed dynamics. Experiments by Frauenfelder and colleagues using Mossbauer and neutron scattering techniques demonstrated that internal fluctuations of proteins are linked to the dynamics of the surrounding solvent [12], [13], leading to the idea that protein dynamics is ‘slaved’ by [13]–[17] (or coupled to [18]) solvent dynamics. Many other experimental and theoretical studies arrived at similar conclusions [19]–[24]. However, it is less clear how buried solvent molecules might modulate an allosteric coupling between spatially distant regions in multi-domain or monomeric proteins [25]–[28]. A better understanding of how protein-bound waters modulate coupled motions and allostery can lead to new strategies for ligand and protein design.

Water molecules reported in crystallographic structures, particularly those located at domain boundaries, interfacial regions or near active sites, often have a structural or functional role [29]–[34]. In contrast, the role of buried waters located far away from the active or ligand-binding site of a monomeric protein is not always obvious. In some cases, these waters may participate in long-range hydrogen bond networks that connect distal protein segments to functionally important regions. Recent reports indicate that this applies to H-ras [35]–[36], a close homologue of the subject of this study, K-ras. Both H- and K-ras belong to the Ras family of molecular switches that play a central role in a variety of signaling pathways [37]–[39]. Ras is turned on when bound to guanosine triphosphate (GTP) and off when bound to guanosine diphosphate (GDP) [40]. The population of the ‘on’ and ‘off’ states is regulated by guanine nucleotide exchange factors (GEFs), which increase the dissociation rate of GDP, and GTPase-activating proteins (GAPs), which accelerate the slow intrinsic rate of GTP hydrolysis [39], [41], [42]. Oncogenic mutations that impair intrinsic Ras function and/or GAP action are found in ∼15% of all human tumors and in up to 90% of cases in specific tumor types [38], [43], [44].

**Figure 1:**
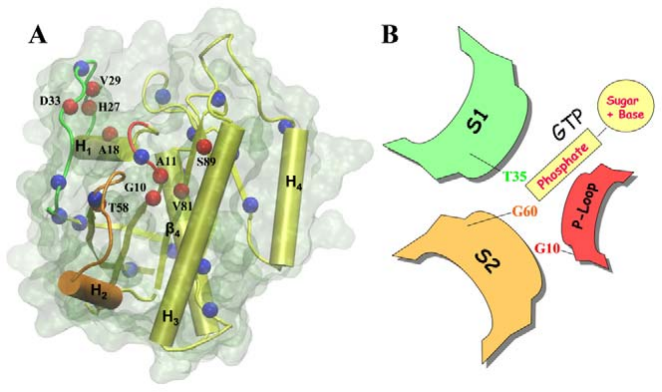
Conserved waters in Ras crystal structures, and functionally key regions. (A) Backbone nitrogen atoms of the Q61H K-ras x-ray structure were used to highlight protein sites occupied by one or more water molecules in >70% (red) and 50–70% (blue) of the available Ras crystal structures (the analyzed structures are listed in Table S1). P-loop (residues 10–17), switch1 (residues 25–40) and switch2 (residues 57–75) are colored in red, green and orange, respectively. The rest of the protein is in yellow cartoon and the molecular surface in transparent light green. (B) Schematic illustration of some of the key residues in the functionally important P-loop and switch regions.

The catalytic domain of K-ras is bi-lobal, with lobe1 (residues 1–86), but not lobe2 (residues 87–167), being evolutionarily conserved among the Ras family [45]–[48]. The major structural difference between the GDP- and GTP-bound forms involves two switch regions located in lobe1 (S_1_: residues 25–40 and S_2_: 57–75; see Fig. 1). Among several key residues known to have distinct interactions in the inactive and active forms are T35 on S_1_ and G60 on S_2_ (See Fig. 1B), which interact with Mg^2+^ and the γ-phosphate of GTP; GTP hydrolysis leads to the loss of these interactions and relaxation of the switches to an open conformation [39], [49]. Furthermore, a recent ^31^P-NMR [50], [51] study indicated the presence of two conformational states in GTP-bound H-ras, state_1_ and state_2_ [52]. The two states are characterized by different chemical shifts in the α- and γ-phosphorous atoms of the GTP and by the ability of state_2_ to interact with effector proteins while state_1_ is stabilized by GEFs [51].

To investigate the role of buried water molecules on the distribution of functionally relevant conformational states of K-ras, we analyzed all available Ras x-ray structures and conformers derived from seven sets of twenty explicit solvent multi-copy MD simulations of Q61H K-ras. The simulations were performed with and without restraints on selected water molecules and protein segments, as well as in the presence and absence of two conserved protein-bound water molecules. Using a pair of simple, previously uncharacterized, reaction coordinates we grouped both the experimental and simulated structures into active GTP-bound, intermediate-like GTP-bound, inactive GDP-bound and nucleotide-free conformational states. We describe the effect of selected protein-bound waters on the distribution of these states.

## Results/Discussion

Analysis of available Ras crystal structures (see Methods) yielded a number of conserved water molecules around the switch regions and the P-loop (Fig. 1A). To evaluate the structural and functional significance of these waters, we carried out a systematic study based on seven sets of multi-copy MD simulations (Table 1). In these independent simulations, crystal waters W_3_ and W_4_ were either (i) unaffected, (ii–iii) individually restrained, (iv) simultaneously replaced by dummy molecules, (v) both removed at the start of the simulations or (vi–vii) their gating protein segments S_2_ and H_3_ harmonically restrained in separate simulations.

**Table 1:** Summary of the simulations.

Simulation	# of copies, length (ns)	Remarks
*F*	3, 100	All crystal waters kept, no restraints
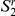	3, 100	Switch-2 Cα atoms restrained
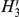	3, 100	Helix-3 Cα atoms restrained
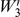	3, 60	W_3_ oxygen atom restrained
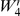	3, 60	W_4_ oxygen atom restrained
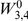	3, 100	No partial charges on W_3_ and W_4_
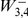	2, 100	W_3_ and W_4_ removed

The aggregate simulation time is 1.76 µs. A force constant of 10 kcal/mol/Å^2^ was used for the restraints.

### Identification of conserved crystallographic water molecules

As of 2010, the protein data bank has 53 entries of Ras crystal structures comprised of 65 chains (see supplementary Table S1). Of these, 45 chains with a resolution of 2.8 Å or better contain crystallographic waters. Five water molecules were found conserved in over 70% of these structures. Fig. 1A shows the location of these waters in the protein based on their nearest neighbor, which is defined as the nearest of any backbone nitrogen or oxygen atom that is within 3.5 Å of a water oxygen atom. Two of the five water molecules are well-known for their interaction with the bound nucleotide [53]. However, little attention has been given to the remaining three waters, despite their apparent role in stabilizing the functionally critical nucleotide binding switches and the P-loop (Fig. 1A). These include W_1_ coordinated by H27 and V29 at S_1_ and A18 at helix H_1_, W_3_ interacting with G10 at the P-loop as well as side-chain atoms of T58 and R68 at S_2_, and W_4_ which interacts with backbone atoms of A11 at the P-loop, V81 at β_4_ and S89 at H_3_. The average reported B-factors for W_3_ and W_4_ are 22 and 19 Å^2^, suggesting limited thermal fluctuation. The average backbone solvent accessible surface areas (SASA) of their nearest neighbors G10 and A11 are 2.2 and 1.2 Å^2^. In contrast, W_1_ is relatively dynamic with a B-factor of 24 Å^2^, and surface exposed with a backbone SASA of 2.9 Å^2^ for its nearest neighbor A18. W_3_ and W_4_ are curiously missing in several Ras structures with mutations at position 12 (P-loop, e.g., PDB: 621P), 38 (effector binding loop, e.g., PDB: 221P), and 61 (S_2_, PDB: 721P). In addition, W_3_ (but not W_4_) is missing in a G12D variant (PDB: 1AGP) and a Ras-Sos complex (PDB: 1NVW), whereas W_4_ is missing in G12R (PDB: 421P), another Ras-Sos complex (PDB: 1HE8) and a Ras-PI3K complex (PDB: 1BKD). It is important to note that oncogenic mutations frequently occur at positions 12 and 61, and mutations at position 38 often impede effector binding. Moreover, W_4_ is located at the interface between the two lobes of Ras, connecting the P-loop in lobe1 with H_3_ in lobe2 via the interfacial strand β_4_ (Fig. 1A). These observations prompted us to undertake a systematic MD analysis of the structural and functional roles of these waters.

### Hydration waters in unrestrained MD trajectories of Q61H K-ras

**Figure 2:**
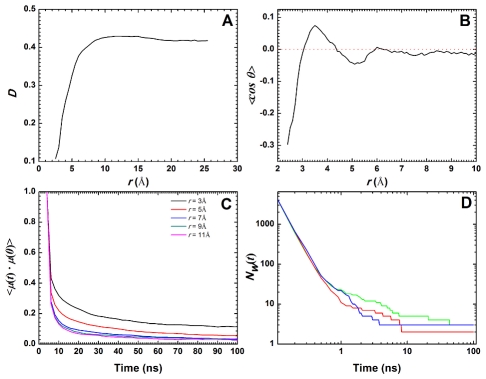
Dynamics of protein-bound waters. Anomalous behavior of hydration waters in the unrestrained simulations (the *F* series in Table 1) are characterized by: (A) the radial profile of the diffusion coefficient, *D*, and (B) the dipole orientation order parameter, *<cosθ>*, as a function of distance *r* from the protein surface. Also shown are the dipole autocorrelation function at different *r* values (C), as well as the survival probability function, *N_w_(t)*, of waters in the first hydration shell (D).

The time- and ensemble-averaged water diffusion coefficient, *D*, (see Methods) calculated as a function of distance from the protein surface, *r*, shows that water molecules within 2.5–5.0 Å of the protein diffuse very slowly (*D*≈0.12–0.33 Å^2^/ps; see Fig. 2A). *D* progressively increases and stabilizes after *r*≈10 Å to ∼0.4 Å^2^/ps, which corresponds to the bulk-water diffusion coefficient of the TIP3P water model used in this work [54]. The reduction in translational motion of the protein-bound waters is coupled with a change in their orientation, as *<cosθ>* ranges between −0.3 and 0.1 in the region 2.4≤*r*≤6 Å before stabilizing near zero (Fig. 2B). Further information about the influence of the protein-solvent interaction on the diffusive property of waters in different hydration shells can be obtained from the autocorrelation function of the electrical dipole moment, *μ*. Plots of this function for *r* values of 3–11 Å at an increment of 2 Å clearly show that the reorientation dynamics of the water dipoles in the first hydration shell (*r* = 3.0 Å) is much slower than those in the second hydration shell (*r* = 5.0 Å), which in turn is much slower than those in bulk (Fig. 2C). Previous studies have shown that protein-solvent interaction, especially hydrogen bonding, is the primary reason for the retarded dynamics of protein-bound waters [55]. The affinity of these hydration waters for the protein can be quantified by their survival probability, *N_w_(t)*. A plot of *N_w_(t)* versus time indicates that up to 6 waters have a survival probability of at least 8–10 ns in the three unrestrained trajectories (Fig. 2D). Of these, 2–3 waters survived for the whole 100 ns duration of the simulations. This indicates that some of the conserved crystal waters described in the previous section are stably bound to the protein in solution and can be considered an integral part of the protein structure. The stability of these water molecules is remarkable given the higher diffusive property of TIP3P waters compared with some of the other more popular water models [56]–[59]. In sum, our results about the hydration behavior of Q61H K-ras are consistent with many previous reports on the hydration of other proteins, such as lysozyme [55] and plastocyanins [60].

### Long-residence waters

**Figure 3:**
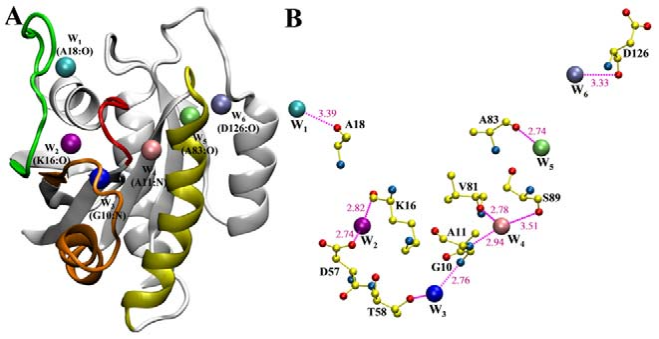
A “belt” of long-residence waters in Ras. (A) Six water molecules (W_1_–W_6_) found to be stably protein-bound during the unrestrained simulations are shown along with their nearest backbone atoms. The protein is in gray except for the P-loop (red), switch1 (green), switch2 (orange), and helix3 (residues 84–104, yellow). (B) Hydrogen-bonding interactions between backbone or side-chain protein atoms and the six water molecules. Since the interactions involving residues T20, H27, V29 and R68 are dynamic, they are not present in this particular snapshot and hence not shown. Color code: carbon, yellow; oxygen, red; nitrogen, blue.

Six water molecules were found to have t_mean_(α)≥1 ns (see eq. 2 and Table 2, Fig. 3A). Each of these waters interacts with 2–3 backbone or side-chain atoms of nearby residues (Fig. 3B). Nearest-neighbor analysis (see Methods) identified A18:O, G10:N, and A11:N as the primary interaction partners of W_1_, W_3_, and W_4_, respectively. These are the same water-binding sites found in the majority of the x-ray structures (Fig. 1A), indicating their preservation during the simulations. The remaining three waters W_2_, W_5_ and W_6_ at sites K16:O, A83:O and D126:O were not present in the starting structure or the majority of the Ras x-ray structures.

**Table 2:** Mean and maximum residence times, and mean square deviation of selected protein-bound waters.

Water	t_mean_(α) (ns)		t_max_(ns)		MSD_tmax_ (Å^2^)	
	*F*	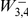	*F*	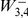	*F*	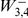
W_1_	17	3	51	85	2.68	0.63
W_2_	11	12	42	76	0.59	0.49
W_3_	3	5	26	26	0.48	0.48
W_4_	41(b)	96	100	100	0.39	0.35
W_5_	2	12	2.4	29	0.63	0.85
W_6_	1.5	11	4	55	1.24	0.57

Only water molecules that are protein-bound in simulations *F* and 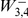 are shown. The mean residence time, t_mean_(α) was calculated using eq.2. t_max_ is the maximum mean residence time at a particular hydration site. The mean square displacement of a water molecule with t_max_ (MSD_tmax_) was calculated using eq. 3.

(b) The relatively small t_mean_(A11:N) is because W_4_ has exchanged and eventually escaped in one of the three simulations, though it was stable for the whole 100 ns in the other two.

W_1_ has a mean residence time at site A18:O (t_mean_(A18:O)) of 17 ns (Table 2) and occupancy of 30% (see Methods). The other interaction partners of W_1_ are the backbone polar atoms of H27 in lobe1 and occasionally A146 in lobe2. The latter interaction often occurs through another water molecule and appears to facilitate hydrogen bonding between A146 carbonyl and the nucleotide base. In this context, it is important to mention that A146T K-ras mutation has been recently implicated in colorectal cancer [61]. W_2_, which bridges the backbone of K16 and the side-chain of D57, has a mean residence time (t_mean_(K16:O)) and occupancy of 11 ns and 20%, respectively. K16 stabilizes the phosphate of the nucleotide [39], while D57 is part of the conserved D_57_TAGQ_61_ motif [62], [63]. W_3_ and W_4_ have occupancies of 72 and 84% at their respective sites G10:N and A11:N. They are tightly bound to these atoms with a mean square displacement (MSD) of 0.48 and 0.39 Å^2^. W_3_ is located near the highly flexible S_2_ and is further stabilized by interactions with the side chain functional groups of T58 and R68. As a result of S_2_'s dynamics, W_3_ frequently exchanges with bulk water (t_mean_(G10:N)≈3 ns), suggesting that water binding at this site is entropically favored. In contrast, the more buried W_4_ rarely exchanges with bulk water (t_mean_(A11:N)≈41 ns), suggesting a strong enthalpic binding that involves accepting a hydrogen bond from A11:N-H and donating to V81:O and S89:O. This internal water thus couples the P-loop to H_3_. Note that the smaller t_mean_(A11:N) is due to the exchange and eventual loss of W_4_ in one of the three trajectories (t_res,j_ = 100 ns in the other two). The non-crystallographic waters W_5_ and W_6_ also have significant t_mean_ (2.0 and 1.5 ns) at their respective sites A83:O and D126:O.

Overall, these six long-residence waters stabilize functionally critical regions, such as the P-loop and the nucleotide binding switches, or physically connect the evolutionarily conserved lobe1 with the variable lobe2. The latter is similar to previous reports on protein-kinase A where several water molecules participate in an extended network of inter-domain interactions [19], [33].

### Novel reaction coordinates to characterize Ras conformational states

To further investigate the functional role of the long-residence waters discussed above, it is important to identify features of the protein that may change with the presence and absence of these waters. In previous reports, we used principal component analysis (PCA)-based collective coordinates to discriminate between active and inactive conformations of Ras [45], [46], [64], [65]. We found that the wild-type GDP- and GTP-bound structures form distinct clusters while mutant structures group into separate clusters that are intermediate between the two major clusters [45], [46]. Though dominated by the switch regions, PCA contains contribution from the overall dynamics of the protein. Since the effect of individual water molecules may be localized near their binding site, we searched for reaction coordinates that only involve protein segments in the vicinity of W_3_ and W_4_. Ideally, such reaction coordinates should also be able to discriminate between the fully active GTP-, less active GTP-, inactive GDP-bound and nucleotide-free conformations. (For brevity, we will refer to these states as the GTP, intermediate, GDP and free state.) The GTP and GDP states are very well characterized and covered in numerous excellent reviews (e.g., [26], [39], [66], [67]). Nucleotide-free conformations, often found complexed with Sos [68], are characterized by wide-open switch conformations. The intermediate state is comparatively less well-characterized, but several structural and biochemical studies have shown that some mutations on S_1_ (e.g., T35S) and S_2_ (e.g., G60A) lead to intermediate GTP-bound structures [62], [63], [69], [70] that are defective in effector recognition [51], [52].

**Figure 4:**
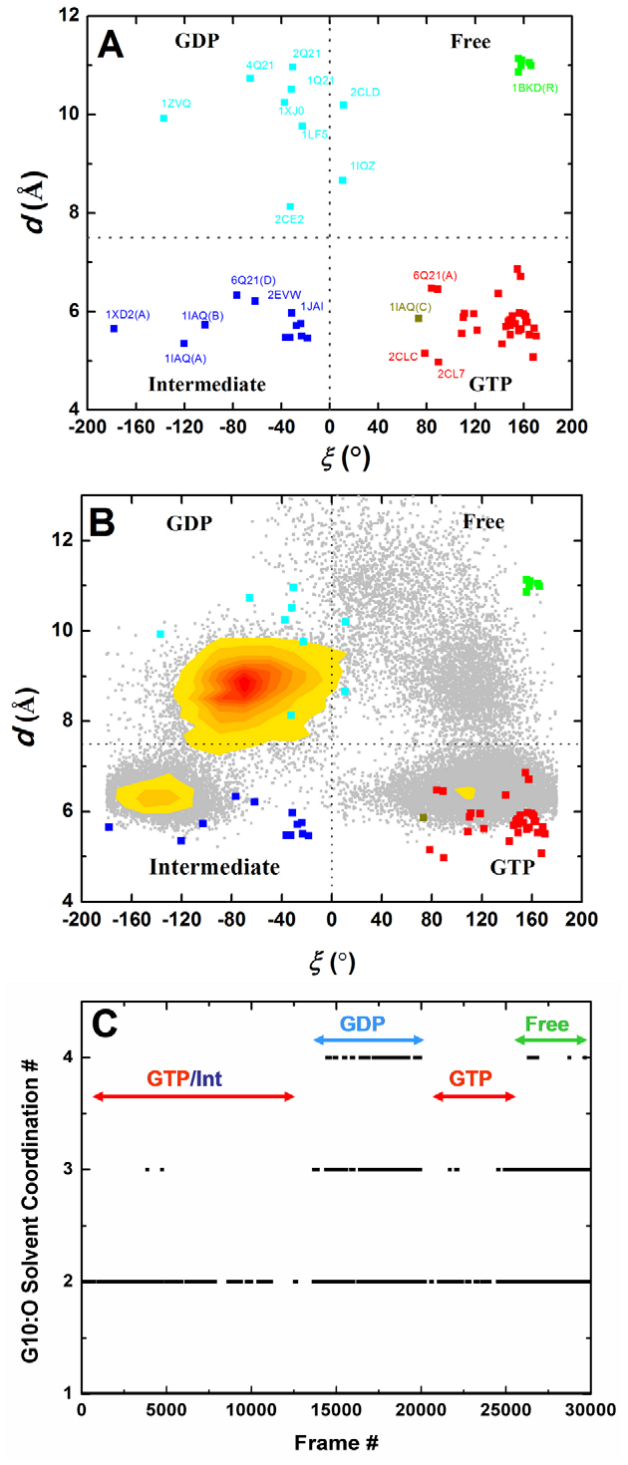
Analysis of crystal and MD-derived Ras conformers. (A) Classification of Ras x-ray structures into GTP (red), intermediate (blue), GDP (cyan) and nucleotide-free (green) states based on the distance (*d*) between the C_α_ atoms of G10 and G60, and the N-C_α_-C-O dihedral of G60 (*ξ*). PDB ids and exceptions are labeled where space permits. (B) Overlay of MD-derived d/ξ values (light gray) onto those derived from the crystal structures. Also shown is a heat map cluster (red is most intense) of conformers in which W_4_ is absent. (C) The time evolution of the water coordination number for G10 carbonyl oxygen. See text for the definition of states.

The adjacent P-loop residues G10 and A11 interact in two different directions via W_3_ and W_4_: with T58 & R68 involving the conserved D_57_TAGQ_61_ motif in lobe1 and with V81 & S89 in lobe2, respectively. Considering the role of G60 in stabilizing the γ-phosphate of GTP via its amide group (Fig. 1B) and the displacement of S_2_ away from the P-loop in the GDP state [39], we reasoned that the distance between the C_α_ atoms of G10 and G60 (*d*) and the G60 N-C_α_-C-O dihedral (*ξ*) may prove useful (Fig. 1B). Indeed, Fig. 4A shows that analysis of the x-ray structures in our data set in terms of *d* led to two groups populated by GTP-bound (*d*≤7.5 Å) and GDP-bound plus nucleotide-free conformers (*d*>7.5 Å). A similar analysis in terms of *ξ* produced two other groupings populated by GDP plus intermediate conformers (*ξ*≤0.0°) on the one hand, and GTP plus nucleotide-free conformers (ξ>0.0°) on the other. A scatter plot of *d* versus *ξ* yielded four distinct clusters populated by structures in the GTP, intermediate, GDP and nucleotide-free state (Fig. 4A). It is remarkable that these simple reaction coordinates enable such a neat discrimination among states, including the intermediate GTP-bound sub-state populated by mutant structures (e.g., A59G, PDB: 1FL0) and few Ras-Sos complexes (e.g., PDB: 1NVX) (see Supplementary Material). The active and inactive clusters largely coincide with the previous PCA results [45], [46], [64], [65], apart from the GDP-bound G12V structure (PDB: 2Q21) that, unlike in the PCA analysis, now clusters with the rest of the inactive structures. This is to be expected because the current classification emphasizes S_2_ while it was the uniquely open S_1_ conformation that separated 2Q21 from the main inactive cluster [45], [46]. Overall, our *d/ξ* plot centered on S_2_ and the P-loop effectively captures the nucleotide dependent conformational dynamics of Ras.

### Protein-bound waters modulate the distribution of Q61H K-ras conformational states

Projection of the MD-derived *d/ξ* values into the *d/ξ* space defined by the crystal structures shows that only 45% (see Methods) of the MD conformers sample from the GTP state (Fig. 4B). Another 25% went to the intermediate state while the remaining conformers are distributed in the GDP and nucleotide-free states. Clearly, Q61H K-ras samples all four conformational states at room temperature and in the presence of GTP, with the active state being the most favored, followed by the intermediate state. This result thus re-enforces our previous finding that Ras exists in different conformational sub-states in solution and predominantly operates via a conformational selection mechanism [64].

Projection of conformers with and without W_3_ at G10:N (Fig. S1, A&B) indicates no correlation with the water-occupancy of this site and the protein conformation. This implies that dynamics of the flexible loop of S_2_ is not directly dependent on W_3_, as one would expect from the interaction of this water with S_2_ residues T58 and R68. Interestingly, however, escape of the distal W_4_, which occurred during one of the three *F* series of trajectories and represents about 17% of the conformers, shifted the equilibrium towards the more open inactive state (Fig. 4B). This is surprising because, unlike W_3_, W_4_ does not directly interact with S_2_ whose motion dominates the inter-state dynamics captured by the *d/ξ* plot. This can be understood by noting that the S_2_ loop around G60 moved away from H_3_ in conformations lacking W_4_, which allowed for W_4_ to escape from its deeply buried position between the two lobes. This resulted in an increased solvation of G10, as shown by the water coordination number of its carbonyl oxygen from 1–2 in the presence of W_4_ to 3–4 in its absence (Fig. 4C). Thus, W_4_ influences the solvation behavior of W_3_'s nearest neighbor even if the dynamics of the two waters is not strictly coupled.

While waters W_1_ and W_6_ do not seem to affect the dynamics of S_2_, entry of W_2_ and W_5_ in the latter half of the simulations resulted in significant changes in the conformational distribution (Fig. S1, C–F). In the presence of W_2_ the intermediate state is favored over the GTP state. In contrast, conformers containing W_5_ preferentially sample the nucleotide-free region.

Taken together, the decoupled dynamics of the protein with some waters, such as W_3_, and its strong coupling with others, such as W_4_, implies that a global master-slave relationship of water and protein dynamics is not always applicable at the level of individual water molecules. The relationship between water and protein motion appears to be modulated by subtle differences in binding sites, such as whether a given structural water is entirely coordinated by the less flexible backbone atoms instead of side chains. Our results also underscore that the dynamics of individual water molecules can influence protein fluctuation locally or at a distance.

### Protein controlled water dynamics and the role of protein-water hydrogen bonding

To assess the role of backbone dynamics on the fluctuation of individual protein-bound waters and evaluate the extent of their coupling, we ran four independent sets of additional simulations.

**Figure 5:**
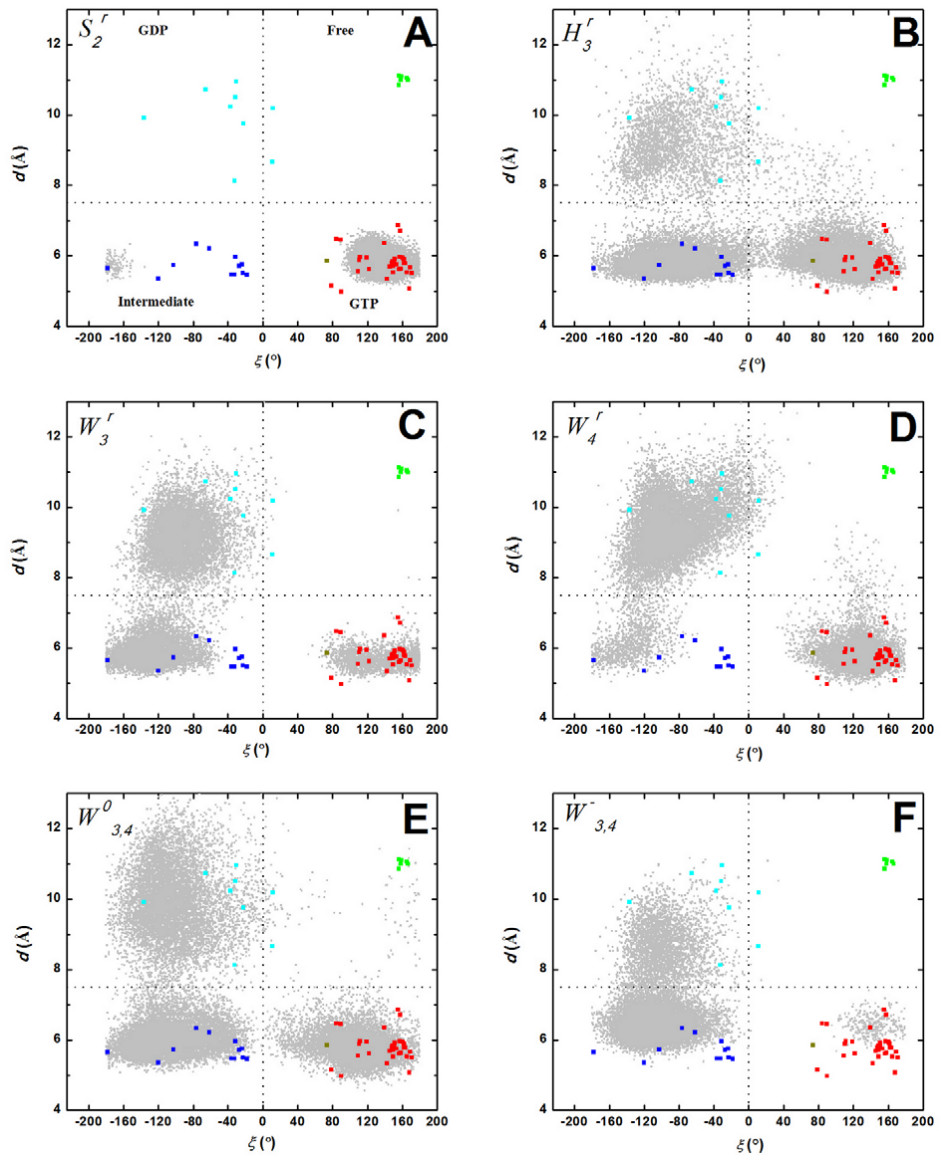
Re-distribution of conformational states by structural waters. *d/ξ* values from MD (gray) are overlaid with those from x-ray structures (symbols) for simulations 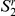 (A); 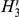 (B); 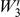 (C); 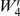 (D); 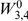 (E); and 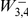 (F). See legend of Fig. 4 for color scheme and Table 1 for simulation names.

1. *Restraining protein segments controlling the dynamics of structural waters* (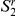 and 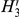): In two sets of three 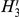 independent runs (each 100 ns-long), a harmonic force constant of 10 kcal/mol/Å^2^ was applied on the C_α_ atoms of S_2_ and H_3_ that gate W_3_ and W_4_, respectively. A *d/ξ* analysis of the 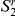 trajectories (Fig. 5A) indicates a restricted sampling in a region confined to the starting structure. W_3_ did not exchange in any of the three 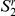 trajectories, consistent with our initial expectation that lack of S_2_ dynamics would abolish exchange of W_3_ with bulk. Thus S_2_ gates the entry and exit of W_3_ even if their dynamics is not strictly concordant (see previous section). W_4_ also remained stable during these simulations, suggesting an indirect stabilization of W_4_ by S_2_. In the 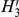 trajectories, the vast majority of the conformers populated the GTP and intermediate states (Fig. 5B). This is consistent with the results of the unrestrained simulations in which these two states were favored by conformers with intact W_4_ (Fig. 4B).
2. *Restraining selected protein-bound waters* (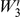 and 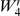): In another two sets of three 60 ns runs, W_3_ and W_4_ were positionally restrained to evaluate the specific role of protein-water hydrogen bonds for the stability and dynamics of the protein and the waters themselves. As expected, the restriction of W_3_ in each of the 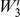 simulations abolished its exchange with bulk even if S_2_ was still dynamic. The inability of W_3_ to undergo rotational dynamics did not allow it to remain hydrogen bonded with the fluctuating protein, which led to the early reorientation of its principal interaction partner G10:N (*ξ*<0, see Fig. 5C). As a result, the intermediate and GDP states were favored by an overwhelming majority (∼87%) of the conformers. W_4_ also remained tightly bound in all of the 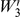 trajectories. Unlike W_3_ whose restriction in the initial position was tolerated by the protein, restraining W_4_ led to an enhanced global displacement of the protein away from W_4_. This happened within 0.7–2.2 ns in each of the 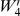 simulations, but it took 2–35 ns for a new water molecule to come in. This lag time allowed the protein to spend a considerable amount of time in the GDP state (Fig. 5D). Taken together, these results show that the dynamics of deeply buried waters that exclusively interact with backbone atoms is intimately linked to the dynamics of the protein, whereas the dynamics of less buried waters partially stabilized by hydrogen bonds with side chains, such as W_3_, is less coupled with the protein motion.
3. *Removal of partial charges in protein-bound waters* (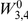): Yet another set of three 100 ns simulations were carried out to further evaluate the apparent link between the rotational dynamics of structural waters, such as W_4_, and their ability to remain hydrogen bonded with the fluctuating protein. In these simulations W_3_ and W_4_ were simultaneously replaced by dummy molecules (i.e., with mass and structure of a water molecule but lack partial charges). During each simulation the uncharged initial waters were quickly replaced (within few nanoseconds) by other water molecules. The quick replacement of the hydrogen bond-deficient waters by those capable of forming hydrogen bonds led to conformational sampling similar to the unrestrained simulations, apart from the absence of conformers that resemble the nucleotide free form (Fig. 5E). These results further highlight the importance of solvating backbone atoms with unsatisfied hydrogen bonds, and emphasize how compounds with defective hydrogen bonding capabilities may not be tolerated at water-binding sites.
4. *Removal of selected protein-bound waters* (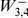): In each of the two 100 ns-long 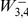 simulations, bulk waters quickly re-entered (during equilibration or within few nanoseconds of the production phase) to take the place of the removed W_3_ and W_4_. However, the vast majority of the resulting conformers (81%) are in the intermediate state, with ∼17% wandering off to the GDP state and a negligible proportion (2%) remaining in the GTP state (Fig. 5F). The trigger for the re-distribution of the conformational states is hard to pinpoint, but it appears that absence of the two waters, especially W_4_, in the beginning of the simulations resulted in the re-orientation of G60 in the S_2_ loop. Another unique feature of these simulations is that the non-crystallographic W_5_ and W_6_ have larger t_mean_ (12 and 11 ns, Table 2) and occupancy (58 and 66%) at sites A83:O and D126:O. Of the two, W_5_ appears to play a particularly important role in conformational sampling, as described below.

### Water-mediated allosteric pathways

**Figure 6:**
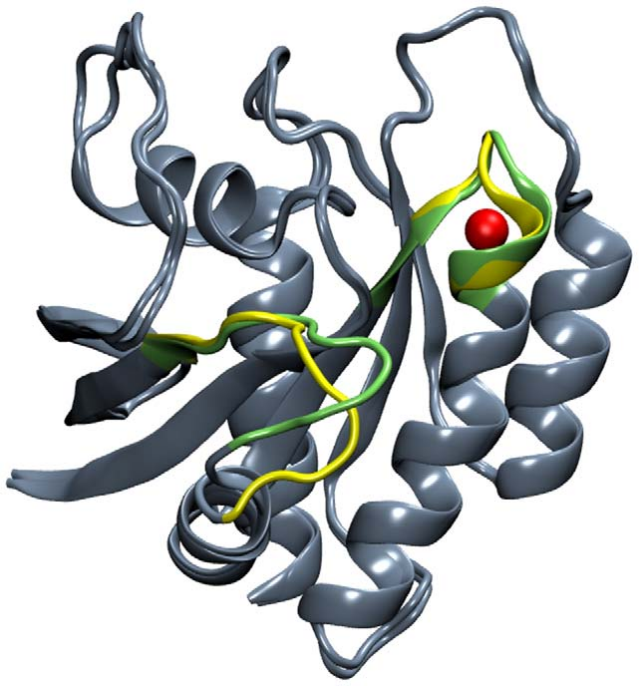
Superimposition of the average structures of conformers with and without W_5_ (red sphere). Major differences are highlighted in green (with W_5_) and yellow (without W_5_), showing allosteric modulation of S_2_ by W_5_ located at the N-terminus of H_3_ across the lobe interface.

In the unrestrained simulations, W_5_-containing conformers (representing 4% of the total) are characterized by *ξ*>0°/*d*>7.5 Å and preferentially populate the nucleotide-free region (Fig. 4B). In contrast, W_5_-containing conformers derived from the 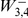 simulations are characterized by *ξ*<0°/*d*<7.5 Å and populate the intermediate state (Fig. 5F). The reason for this discrepancy is not clear, but it may be related to the recently proposed multi-pathway nature of the allosteric effect [71] where an ensemble of states (instead of a single propagation pathway) defines a particular allosteric interaction. In both cases, however, the GTP state is disfavored, and entrance of W_5_ to the N-terminus of H_3_ coincides with a large conformational change of S_2_, as shown by superposing the average structures with and without W_5_ (Fig. 6). As an example, A83:O and S89:OH were ∼3.6 Å apart in the absence of W_5_ but a partial loss of a helical turn at the N-terminus of H_3_ upon the entry of W_5_ led to increased solvation of S89. That this local conformational change at the N-terminus of H_3_ is felt by S_2_ suggests their allosteric coupling, with W_5_ acting as a ligand. Though we are not aware of a report linking S_2_ dynamics with the N-terminus of H_3_, previous studies have found a water-mediated interaction between Y32 at S_1_ and N86 at the N-terminus of H_3_ [35], as well as a correlated motion between S_2_ and the C-terminus of H_3_ [46]. Although the full implication of this result to Ras function warrants further investigation, it underscores the potential of multiple/long simulations to uncover mechanistic insights hidden in crystallographic average structures. Nonetheless, the following observations highlight how water-mediated allostery might underlie the differential sampling of phase space by Q61H K-ras in the presence and absence of specific water molecules.

**Figure 7:**
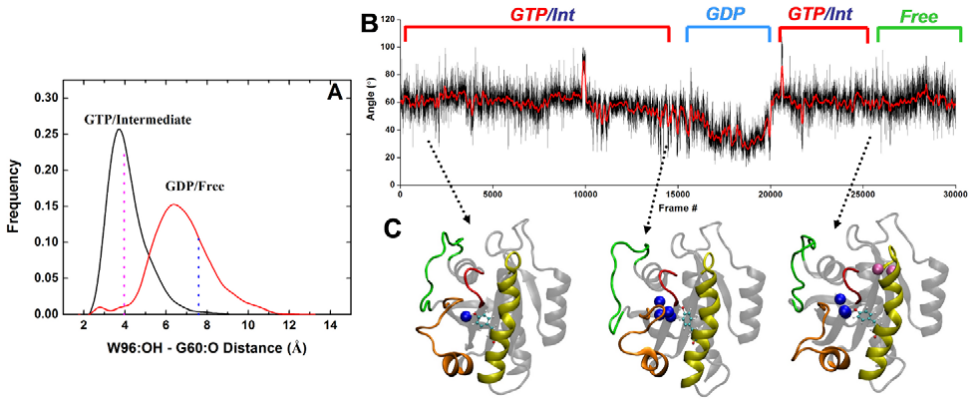
The role of Y96 in a proposed water-mediated allostery. (A) Histogram of the Y96:OH-G60:O distance in the unrestrained simulations plotted separately for conformers having *d* (see Fig. 4) less than (black) and greater than or equal to (red) 7.5 Å. Vertical dashed lines indicate the corresponding values averaged over the GTP- (left) and GDP-bound (right) x-ray structures. (B) Evolution of the angle between vectors connecting C_α_ atoms of S89 and Y96, and C_γ_ and OH of Y96 in the concatenated unrestrained simulations. (C) Representative snapshots highlighting conformers in the GTP, GDP and nucleotide-free states.

Recent studies by Buhrman et al. [35], [36] showed that a conformational change at H_3_/loop7 allosterically modulates S_2_ and thereby positions Q61 for a direct action in GTP hydrolysis. One of the two hydrogen bond networks responsible for the allosteric coupling involves Y96, Q99 and R102. In our simulations Y96 underwent major displacement away from S_2_, thereby increasing the solvent accessibility of key P-loop and S_2_ backbone atoms (Fig. 7). The distribution of the distance between G60:O and Y96:OH (Fig. 7A) shows that the phenoxy group of Y96 is hydrogen bonded, often through a water molecule, with the carbonyl of G60 in the GTP-like conformers (with a mean distance of 3.5 Å for the active and intermediate states). This hydrogen bond is broken as Y96 reorients away from S_2_ in the GDP and nucleotide-free states (average G60:O-Y96:OH distance of 6.5 Å). These mean values closely match the average G60:O-Y96:OH distances obtained from GDP- and GTP-bound x-ray structures (ca. 4.0 and 7.6 Å, Fig. 7A). Consistent with the previously observed positive correlation between the dynamics of S_2_ and H_3_ [46], the reorientation of Y96 is coupled with the displacement of S_2_ away from the P-loop, as suggested by a strong correlation (R = 0.78) between distances *d* and Y96:OH-G60:O. The reorientation of Y96 side chain toward H_3_ was analyzed by the angle between a vector connecting the C_α_ atoms of S89 and Y96 that approximately traces the helical axis, and a vector from the C_γ_ to the OH atoms of Y96 along the plane of the ring (Fig. 7B). The angle decreased from ∼60° in the GTP/intermediate states to as low as 30° in the GDP state. The decrease is accompanied by the expulsion of W_4_ and a significant increase in the water coordination number of G10, i.e., the site of W_3_ binding (Fig. 4C & 7C). Entry of waters such as W_5_ at the N-terminus of H_3_ did not result in a similar Y96 reorientation, but is associated with a larger opening of S_2_ and entry of other solvent molecules.

These observations led us to conclude that the way in which Q61H K-ras samples conformational states, and the population of those states, is modulated in various ways by protein bound water molecules. The observed water-mediated correlated motion among S_2_, the P-loop and H_3_ is very sensitive to perturbation and may be altered by ligands designed to interfere with the inter-lobe communication of Ras proteins.

### Conclusion

The aim of the current work was to investigate the solution stability and role of buried water molecules on the overall dynamics of Q61H K-ras, as well as to assess if crystallographic waters have a role in the inter-lobe communication and allosteric behavior of Ras proteins. To this end, extensive restrained and free MD simulations, involving seven sets of 20 multi-copy runs for a total of 1.76 µs, were carried out in the presence and absence of selected crystallographic water molecules. Analysis of hydration waters indicated that the dynamics of K-ras-bound waters is dramatically different from the bulk behavior. Moreover, the dynamics of several deeply buried long-residence water molecules, such as W_4_, is coupled with the local as well as global motions of the protein. As a result, the protein was able to sample different regions of phase space in the presence and absence of some of these waters. Three water molecules, namely, W_1_ (located in lobe1), W_4_ (at the interface between the lobes) and W_5_ (at lobe2) facilitate the relative motion of residues located at the two lobes of Q61H K-ras. On the other hand, the fluctuation of less buried waters is only loosely coupled with the global motion of the protein even in cases where their exchange is gated by the fluctuation of functionally important protein segments, such as the S_2_ loop.

Our analysis was facilitated by two simple reaction coordinates: distance (*d*) between the C_α_ atoms of G60 at S_2_ and G10 at the P-loop and the N-C_α_-C-O dihedral (*ξ*) of G60. A *d/ξ* scatter plot allowed us to classify the available Ras x-ray structures into active GTP-bound, intermediate GTP-bound, inactive GDP-bound, and nucleotide-free states. We emphasize that the structures classified here as intermediates are deficient in effector binding and contain mutations or are derived from complexes with GEFs. Projection of the MD-derived conformers on the *d/ξ* space derived from the x-ray structures enabled us to associate functionally distinct conformational states with the presence and absence of conserved high-residence water molecules. Moreover, we found a water-mediated correlated motion involving S_2_, P-loop and H_3_. This motion is mediated by specific residues that received little attention in previous studies. Notably, the reorientation of Y96 side chain away from S_2_ in GDP-like structures leads to increased solvation of G10 at the P-loop. The presence and absence of this interaction led to differences in the conformation of S_2_, suggesting a water-mediated modulation of S_2_ by H_3_. Thus, water molecules act as allosteric ligands to induce a population shift in the conformational states of the canonical switches.

## Methods

### Crystal structure analysis

53 x-ray structures (65 chains) of Ras were downloaded from the Protein Data Bank (PDB [72]) and analyzed for their water content, focusing only on waters within the first hydration shell and interacting with the protein backbone, defined by a distance cutoff of 3.5 Å between water oxygen and backbone oxygen or nitrogen atoms. Analysis of the fraction of crystal structures containing one or more water molecules bound to a given protein site identified two particularly interesting waters, W_3_ and W_4_ (see Results and Discussion). These waters were selected for a more detailed analysis by MD.

### Molecular dynamics simulations

The 2.27 Å resolution crystal structure of GTP-bound Q61H K-ras (PDB id: 3GFT) was used to perform seven sets of multi-copy MD simulations (Table 1). An oncogenic mutant of H-ras G12V has been shown to sample a wide range of conformational space [45], [46]. We therefore reasoned that the oncogenic Q61H K-ras, the only available K-ras structure, may also sample a large conformational space during classical MD simulation. In the three simulations named *F* (i.e., free), all crystal waters were kept and no restraints were applied. In simulations 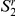 and 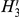, restraints were applied (force constant *k* = 10 kcal/mol/Å^2^) on the C_α_ atoms of switch 2 (S_2_: residues 57–75) or part of helix 3 (H_3_: residues 87–95). Using the same force constant, conserved waters W_3_ and W_4_ were positionally restrained in simulations 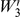 and 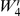, their partial charges were removed in simulations 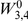 and they were excluded at the start of the 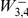 simulations (Table 1). In each case, the system setup involved assignment of charges assuming neutral pH (D, E and C-terminus de-protonated and K, R and N-terminus protonated). The protein was solvated in a cubic box of side 60.4 Å containing TIP3 waters, allowing a minimum of 10 Å distance between the edge of the box and the protein. The system was neutralized by adding 12 Na^+^ ions, and an additional 30 Na^+^ and 30 Cl^−^ ions were added to achieve a 150 mM ionic strength. Energy minimization was then carried out for 2000 steps with the protein heavy atoms fixed and for another 5000 steps with all atoms set free. During the initial 200 ps equilibration, a harmonic restraint of *k* = 4 kcal/mol/Å^2^ was applied on the C_α_ atoms, which was then progressively reduced by 1 kcal/mol/Å^2^ every 100 ps. All equilibration steps used a 1 fs time step, which was increased to 2 fs in the production phase with SHAKE [73] applied to covalent bonds involving hydrogens. The temperature of the system was maintained at the physiological value of 310 K using Langevin dynamics with a damping coefficient of 2 ps^−1^. The Nose-Hoover Langevin piston method was used to maintain constant pressure at 1atm with a piston period of 100 fs and decay time of 50 fs. The short-range van der Waals interactions were switched off gradually between 8.5 and 10 Å with a 12 Å cutoff used for non-bonded list updates. Long-range electrostatic interactions were calculated using the Particle Mesh Ewald (PME) method [74]. Each simulation was run for either 60 or 100 ns, yielding an aggregate simulation time of 1.76 µs. All simulations were performed with the NAMD program [75] and the CHARMM27 force field [76].

### Identification and characterization of hydration waters

For the purpose of this work, hydration waters are defined as waters whose oxygen atom is within 3.5 Å of any non-hydrogen protein atom.

#### Water dynamics

The dynamics of the water molecules that hydrate the protein was characterized in terms of their survival probability (*N_w_(t)*), diffusion coefficient (*D*), dipole (*μ*) orientation order parameter (*<cosθ>*), and dipole autocorrelation function (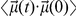). For each water molecule *j*, *N_w_(t)* was calculated based on the conditional probability *P_j_(t_n_,t)*, where *P_j_(t_n_,t)* is 1 if the *j* ^th^ water molecule is within 3.5 Å of any protein backbone atom between time *t_n_* and *t_n_+t*, and 0 otherwise (see ref. [55]). *N_w_(t)* is the sum over *P_j_(t_n_,t)*, which is maximal at the start of the simulation (small *t*) and gives the number of waters permanently attached to the protein at *t* equal to simulation length. The profile of *D* as a function of distance *r* from the protein surface was calculated by a finite-difference method previously described by Makarov *et al.* [77]. *θ* is the angle between a vector along a water molecule's electrical dipole and a vector connecting the center of mass of the protein to the oxygen atom of the same water molecule. The resulting *cosθ* values were averaged over the number of water molecules within a distance *r* from the protein surface, which ranged between 2.5 and 10 Å with an increment of 0.5 Å. *μ* was defined by the vector between a water oxygen atom and the mid point of the distance between its hydrogen atoms. The autocorrelation function 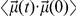 was computed as described in refs. [60], [78].

#### Identification of waters with long-residence times

Garcia and colleagues [79], [80] suggested that the probability that a bulk water molecule has a coordination number (CN) less than 4 for longer than a few picoseconds is negligible. Following this approach, the residence time of water *j*, t_res,j_, was determined by summing over the entire number of frames (N_F_) in which its CN is consecutively less than 4 for a period *t_n,j_* greater than 1 ns:
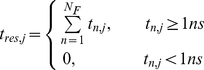
(1)


Water molecules with t_res,j_≥10 ns were defined to be long-resident. Note that these waters may interact with different regions of the protein at different times. To identify protein sites preferred by these waters, we defined the nearest neighbor, *α*, that most frequently interacts with long-residence water *j* based on a distance criterion of 3.5 Å between the water oxygen and a protein backbone nitrogen or oxygen atom. Then, considering those waters that continuously occupy site *α* for at least 1 ns (t_j_(α)≥1 ns), the mean residence time of waters at that site, t_mean_(α), was quantified as,
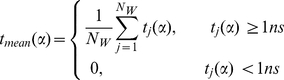
(2)where N_w_ is the total number of water molecules occupying site *α* during the simulations. The maximum residence time, t_max_, is the longest uninterrupted period of time the *j^th^* water molecule occupies site *α*. The mean square displacement (MSD) of a water molecule with t_max_ (W_tmax_) is,
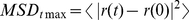
(3)where *r(0)* corresponds to the initial position and *r(t)* the instantaneous position of W_tmax_. Finally, the percent water-occupancy of a given protein site was obtained from the fraction of frames (sampled every 10 ps) that have a water molecule bound to that site.

## Supporting Information

Figure S1 Projection of simulated conformers in the presence of W_3_ (A), W_2_ (C), W_5_ (E) and in the absence of W_3_ (B), W_2_ (D), and W_5_ (F) on *d/ξ* scatter plot derived from crystal structures.
(DOC)

Table S1 A list of PDB structures analyzed.
(DOC)
