# Supplementary material for: The Role of Conserved Waters in Conformational Transitions of Q61H K-ras
Source: PLoS Comput Biol. 2012 Feb 16;8(2):e1002394. doi: 10.1371/journal.pcbi.1002394 (PMC3280954; doi:10.1371/journal.pcbi.1002394)

Supplemental Data

The role of conserved waters in conformational transitions of Q61H K-ras

Priyanka Prakash^1^, Abdallah Sayyed-Ahmad^1^ and Alemayehu Gorfe^1,2^*

University of Texas Health Science Center at Houston, **^1^**Department of Integrative Biology and Pharmacology and **^2^**Center for Membrane Biology, 6431 Fannin St., Houston, Texas 77030

* Corresponding author: Tel: 713-500-7538; Fax: 713-500-7444; E-mail: [Alemayehu.G.Abebe@uth.tmc.edu](mailto:Alemayehu.G.Abebe@uth.tmc.edu)

Keywords:

Molecular dynamics, structural waters, allostery, dynamic coupling, conformational states

Running title:

Role of conserved waters in Ras dynamics

Current Address: ^#^Physics Department, Birzeit University, Birzeit, West Bank.

**Figure S1:** Projection of simulated conformers in the presence of W_3_ (A), W_2_ (C), W_5_ (E) and in the absence of W_3_ (B), W_2_ (D), and W_5_ (F) on *d/ξ* scatter plot derived from crystal structures.


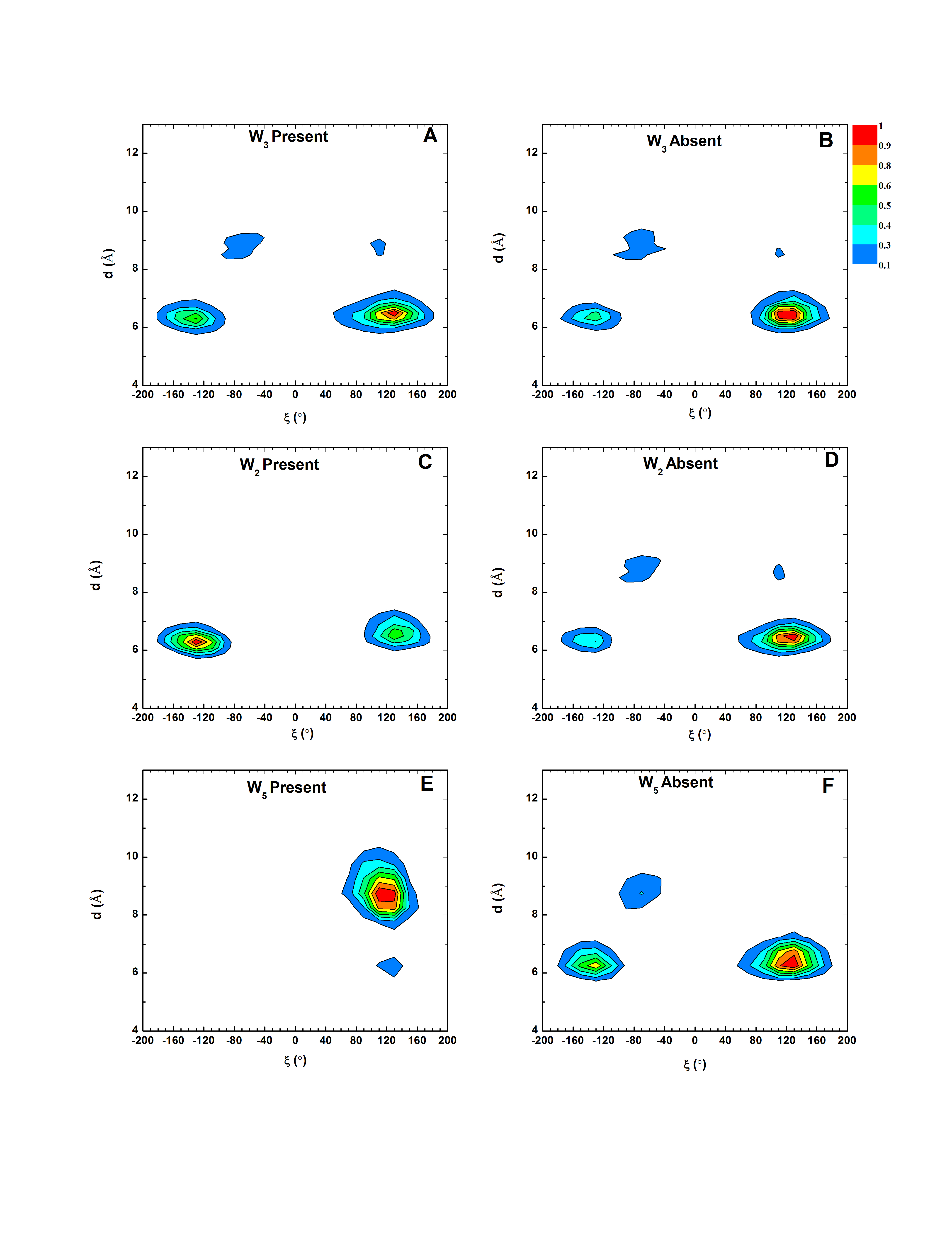

Supplement: Figure S1 — Projection of simulated conformers in the presence of W3 (A), W2 (C), W5 (E) and in the absence of W3 (B), W2 (D), and W5 (F) on d/ξ scatter plot derived from crystal structures. (DOC) [file pcbi.1002394.s001.doc]
