# Supplementary material for: The Role of Conserved Waters in Conformational Transitions of Q61H K-ras
Source: PLoS Comput Biol. 2012 Feb 16;8(2):e1002394. doi: 10.1371/journal.pcbi.1002394 (PMC3280954; doi:10.1371/journal.pcbi.1002394)
Supplement: Table S1 — A list of PDB structures analyzed. (DOC) [file pcbi.1002394.s002.doc]

Supplemental Data

The role of conserved waters in conformational transitions of Q61H K-ras

Priyanka Prakash^1^, Abdallah Sayyed-Ahmad^1^ and Alemayehu Gorfe^1,2^*

University of Texas Health Science Center at Houston, **^1^**Department of Integrative Biology and Pharmacology and **^2^**Center for Membrane Biology, 6431 Fannin St., Houston, Texas 77030

* Corresponding author: Tel: 713-500-7538; Fax: 713-500-7444; E-mail: [Alemayehu.G.Abebe@uth.tmc.edu](mailto:Alemayehu.G.Abebe@uth.tmc.edu)

Keywords:

Molecular dynamics, structural waters, allostery, dynamic coupling, conformational states

Running title:

Role of conserved waters in Ras dynamics

Current Address: ^#^Physics Department, Birzeit University, Birzeit, West Bank.

**Table S1: The analyzed PDB Structures**

| **PDB** | **Resolution (Å)** | **Chain** | **ξ (º)** | **d (Å)** | **Ligand** | **Remarks** | **W_3_, W_4_ ^*^** |
| --- | --- | --- | --- | --- | --- | --- | --- |
| 1IAQ | 2.90 | A | -120.22 | 5.35 | GNP | T35S; no water |  |
| 1IAQ | 2.90 | B | -102.85 | 5.73 | GNP |  |  |
| 1JAI | 1.80 | A | -31.4 | 5.97 | GCP | G12P |  |
| 1LF0 | 1.70 | A | -27.39 | 5.71 | GNP | A59G |  |
| 1LF5 | 1.70 | A | -22.5 | 9.76 | GDP |  |  |
| 1NVU | 2.20 | Q | -18.33 | 5.46 | GTP | Ras-SOS |  |
| 1NVX | 3.20 | Q | -32.75 | 5.47 | GTP | Ras-SOS; no water |  |
| 1PLK | 2.80 | A | -148.79 | 6.34 | GTP | No water |  |
| 1PLL | 2.80 | A | -153.3 | 6.29 | GDP | No water |  |
| 1Q21 | 2.20 | A | -31.7 | 10.51 | GDP |  |  |
| 1XCM | 1.84 | A | -23.8 | 5.75 | GNP | G60A |  |
| 1XD2 | 2.70 | A | -178.04 | 5.65 | GDP | Ras-SOS; no water |  |
| 1XJ0 | 1.70 | A | -37.28 | 10.24 | GDP | G60A |  |
| 1ZVQ | 2.00 | A | -136.92 | 9.92 | GDP | G60A |  |
| 1ZW6 | 1.50 | A | -36.22 | 5.47 | GNP | Q61G |  |
| 2CE2 | 1.00 | X | -32.43 | 8.13 | XY2 | No water |  |
| 2CL0 | 1.80 | X | -23.09 | 5.5 | XY2 | Y32C; C118S |  |
| 2EVW | 1.05 | X | -61.58 | 6.21 | XY2 | Y32C; C118S |  |
| 2Q21 | 2.20 | A | -30.56 | 10.96 | GDP |  |  |
| 4Q21 | 2.00 | A | -65.61 | 10.73 | GDP |  |  |
| 6Q21 | 1.95 | D | -76.68 | 6.33 | GCP |  |  |
| 121P | 1.54 | A | 150.92 | 5.91 | GCP |  |  |
| 1AGP | 2.30 | A | 147.65 | 5.79 | GNP | G12D |  |
| 1BKD | 2.80 | R | 158.02 | 10.99 | - | Ras-SOS |  |
| 1CLU | 1.70 | A | 157.66 | 6.71 | DBG |  |  |
| 1CTQ | 1.26 | A | 161.94 | 5.79 | GNP |  |  |
| 1GNP | 2.70 | A | 156.16 | 5.61 | AGN | No water |  |
| 1GNQ | 2.50 | A | 89.22 | 6.45 | CAG | No water |  |
| 1GNR | 1.85 | A | 111.29 | 5.96 | CAG |  |  |
| 1HE8 | 3.00 | B | 150.67 | 5.84 | GNP | Complex PI3K |  |
| 1IAQ | 2.90 | C | 73.47 | 5.86 | GNP | T35S; no water |  |
| 1IOZ | 2.00 | A | 10.71 | 8.66 | GDP |  |  |
| 1JAH | 1.80 | A | 168.87 | 5.66 | GCP |  |  |
| 1K8R | 3.00 | A | 156.76 | 5.97 | GNP | No water |  |
| 1LFD | 2.10 | A | 165.23 |  | GNP |  |  |
| 1LFD | 2.10 | C | 149.9 |  | 149.9 |  |  |
| 1NVU | 2.20 | R | 155.8 | 10.86 | - |  |  |
| 1NVV | 2.18 | Q | 145.53 | 5.69 | GNP | Ras-SOS |  |
| 1NVV | 2.18 | R | 166.14 | 10.99 | - | Ras-SOS |  |
| 1NVW | 2.70 | Q | 142.26 | 5.34 | GNP | Ras-SOS |  |
| 1NVW | 2.70 | R | 155.92 | 11.13 | - | Ras-SOS |  |
| 1NVX | 3.20 | R | 164.92 | 11.05 | - | No water |  |
| 1P2S | 2.45 | A | 153.32 | 5.75 | GNP |  |  |
| 1P2T | 2.00 | A | 161.94 | 5.89 | GNP |  |  |
| 1P2U | 2.00 | A | 161.17 | 5.91 | GNP |  |  |
| 1PLJ | 2.80 | A | 139.27 | 6.36 | CAG | No water |  |
| 1QRA | 1.60 | A | 160.12 | 5.95 | GTP |  |  |
| 1RVD | 1.90 | A | 155.07 | 6.86 | DBG |  |  |
| 1WQ1 | 2.50 | R | 164.81 | 5.53 | AF3 | Ras-RasGAP |  |
| 1XD2 | 2.70 | B | 158.64 | 11.1 | - | Ras-SOS, no water |  |
| 221P | 2.30 | A | 118.9 | 5.95 | GNP | D30E |  |
| 2C5L | 1.90 | A | 148.63 | 5.83 | GOL |  |  |
| 2C5L | 1.90 | B | 149.2 | 5.71 | GOL |  |  |
| 2CL6 | 1.24 | X | 149.14 | 5.53 | XY2 | Y32C; C118S; No water |  |
| 2CL7 | 1.25 | X | 89.72 | 4.97 | GTP | Y32C; C118S; No water |  |
| 2CLC | 1.30 | X | 78.6 | 5.15 | GTP | Y32C; C118S; No water |  |
| 2CLD | 1.22 | X | 11.32 | 10.19 | GDP | No water |  |
| 421P | 2.20 | A | 109.05 | 5.55 | GNP | G12R |  |
| 521P | 2.60 | A | 167.95 | 5.07 | GTP | G12V; No water |  |
| 621P | 2.40 | A | 110.33 | 5.88 | GNP | G12H |  |
| 6Q21 | 1.95 | A | 84 | 6.47 | GCP |  |  |
| 6Q21 | 1.95 | B | 170.77 | 5.5 | GCP |  |  |
| 6Q21 | 1.95 | C | 158.06 | 5.64 | GCP |  |  |
| 721P | 2.00 | A | 121.85 | 5.62 | GNP | Q61L |  |
| 821P | 1.50 | A | 163.11 | 5.78 | GNP | G12P |  |

*Filled circles show the presence and empty circle the absence of a water molecule labeled 3 or 4.
